# Comparative evaluation of drug information leaflets for non-steroidal anti-inflammatory drugs in Palestine: local versus imported products

**DOI:** 10.1186/s12913-019-4754-1

**Published:** 2019-11-27

**Authors:** Dina A. Arandy, Maysa W. Abu-Hashia, Bahaa M. Al-hroub, Sandra A. Qatmosh, Amer A. Koni, Baraa G. Qeeno, Samah W. Al-Jabi, Sa’ed H. Zyoud

**Affiliations:** 1 PharmD program, College of Medicine and Health Sciences, An-Najah National University, Nablus, 44839 Palestine; 2 Department of Clinical and Community Pharmacy, College of Medicine and Health Sciences, An-Najah National University, Nablus, 44839 Palestine; 3 Clinical Research Centre, An-Najah National University Hospital, Nablus, 44839 Palestine

**Keywords:** Patient package insert, Non-steroidal anti-inflammatory drugs, Palestine

## Abstract

**Background:**

Drug information leaflets (DILs) are written for patients and health care providers to show how to use the medications safely and effectively, in order to reach the required therapeutics outcomes. This comparative study was conducted to evaluate various DILs of non-steroidal anti-inflammatory drugs (NSAIDs) that are produced in Palestine, along with their imported equivalents.

**Methods:**

Thirty-five DILs of NSAIDs were analyzed and evaluated in a cross-sectional comparative study. Thirty-one statements were obtained from literature and used; evaluation was performed on basis of both any presence or absence of these statements in the leaflets. 23 of the 31 statements that were available in both local and imported DILs were also evaluated in terms of total word-counts: the median (interquartile range) word-count for each statement was determined separately for the two groups and then compared. For the remaining 8 statements, this was not performed,either because they were not present in any leaflet, or because counting the number of words would not be meaningful.

**Results:**

A total of 35 DILs for nine different active ingredients of NSAIDs were analyzed. In 97% of leaflets, “Instructions to convert medication into liquid forms” were missing and 94% did not provide any information about “Pharmacokinetics”. 83% of DILs provided no information about “Mechanism of action” and 74% did not mention any reliable references. 66% of the analyzed inserts did not include any instruction about the possibility of a tablet splitting. And in 63%, the “Date of last revision” was missing. Further, “Duration of using” and “Inactive ingredients” were not found in 51% of leaflets. In terms of word-count, the related sections of the 23 selected criteria were expressed with more words by imported leaflets compared with the local ones, significant differences were found in 12 categories, the highest significance of > 42.4-fold difference was found in “Geriatric considerations” category whereas 1.4-fold difference was found in “Shelf life,” being the lowest one.

**Conclusions:**

This study shows that local products provide less information than imported products, so we recommend that appropriate measurements be taken by both Palestinian authorities and manufacturers to improve both quantity and quality of local DILs.

## Background

Drug information leaflets (DILs) are package inserts, providing information for safe and effective medication use, are based on specific regulatory guidelines [1, 2]. DILs should be an unambiguously-written information source about our medications, not only for health care professionals but also for any car givers and, of course, the patients [3]. They should be correct, comprehensive, and up to date, to minimize the incidence of errors during the handling or the taking these medications [4]. DILs should contain information that enhances a patient’s awareness towards optimal medication use, ensuring safety and efficacy to achieve the desired therapeutic plan [5]. It’s been seen that the quantity and quality of DILS information can influence the patients’ compliance and therefore give a satisfactory result [6]. In a systematic review of the role and value of written information for patients about individual medicines, Grime, et al. [7], reported that patients can get confused due to inconsistent DILs information, resulting in a decreased patient medication compliance. Straightforward DILs information plays an important role in helping patients to follow medication instructions properly.

Several studies have shown that availability of information about non-steroidal anti-inflammatory drugs (NSAIDs) to patients and health care providers improves patients’ knowledge about medications they use, enhances benefits and reduces risks in treatment [8–15]. A study in Thailand was undertaken in order to determine information content and availability of NSAIDs leaflets. In that survey, the leaflets of the original drug manufacturers’ products contained more information than Palestine-produced ones. Furthermore, no leaflet mentioned the required warnings that are specified by Thai regulations [16].

The Palestinian Ministry of Health (MOH) requires that medication inserts be bilingual, in both Arabic and English, but does not give any recommendations about specific DIL designs, the quality of information or length of text [17–19]. It was noted that DILs of Palestinian-marketed medications contained incomplete and limited information compared with their counterparts marketed in other countries [5, 20–22]. Although some research has been done of the DILs of medications available in Palestine [5, 20], no study compared the the information provided by local and imported NSAIDS. The reasons that NSAIDs were selected in this study is that they are among the most commonly dispensed medications in the Palestinian pharmacies, and these medications are usually dispensed as self-medications or sold over-the-counter (OTC) [23–25]. Also, these medications are known to cause serious adverse effects, so, in the light of this, the DILs of NSAIDs medications should provide a high quality information to the health-care providers and patients [26, 27]. We performed this study to evaluate and compare the DILs of local and imported NSAID products available in the community pharmacies in Palestine. It is hoped that this research will contribute to a deeper understanding of information gaps in DILs.

## Methods

### Study design

A cross-sectional comparative study was used to assess the quantity and quality of DILs information of NSAIDs.

### DILs selection

Each non-steroidal anti inflammatory active ingredient that included in this study was produced by both a local and a foreign company. Furthermore, all of its alternatives were equivalent pharmaceutically and made of a uniform strength, registered with the Palestinian MOH, and available in the local market; availability was checked by asking 20 different local pharmacies distributed in different governorates of West Bank-Palestine including Hebron, Jenin, Nablus and Ramallah. There are around 1000 community pharmacies in West Bank-Palestine distributed in 11 Governorates [28]. Four governorates were selected randomly to represent the northern, central, and southern regions.

Medications that can be used as anti-platelets; which contain acetylsalicylic acid or dosage forms other than oral such as injections or topical preparations were excluded, as most of these have either fewer side effects or are administered under a health’s professional direct intervention. According to the above selection and exclusion criteria, 9 different NSAIDs were included in this study: Ibuprofen, Indomethacin, Piroxicam, Naproxen, Naproxen Sodium, Diclofenac Sodium, Diclofenac Potassium, Etoricoxib, and Celecoxib. The selected NSAIDs were available in the Palestinian West Bank market as 35 products (*n* = 18 imported products; *n* = 17 Palestinian-produced medicines), all that are available in the market; (Table 1). The 35 package inserts for these products were collected from different pharmacies in Palestine on request.

**Table 1:** list of nine active ingredients of NSAIDs included in our study with their local and imported trade names

Active ingredient	Local products	Imported products
Celecoxib	Celex®,Coxib®	Celecox®, Celebra®
Etoricoxib	Tericox®, Etoflam®	Arcoxia®
Diclofenac Na+	Diclofen®, Rufenal®, Voryn®	Betaren®, Swiss Relief®, Voltaren®
Diclofenac K+	Anaflam®, Joflam®, Toleran®	Cataflam®
Ibuprofen	Isofen®,Trufen® Ultrafen®	(Adex, Adex Forte)®,Advil Liqui-gel®, Artofen®,Ibufen®, Nurofen forte®
Indomethacin	Indocaps®,Indolin®	Indovis®
Piroxicam	Pirox®	Brexin®
Naproxen	Naprex®	Naxyn®
Naproxen Na+	Naproxan®	Narocin®, Point®

### DILs assessment and evaluation

The comparative evaluation was done in two ways. First, a scoring method was carried out based on presence/absence of 31 specific items of information (either in the DILs’ obvious section categories or, less obviously, as extractable subcategories), which are: brand name, active ingredient, inactive ingredients (excipients), therapeutic class, mechanism of action, indications, drug dose, duration of using, missing dose, maximum dose, directions for use, overdose and management, warning and precautions, effect on ability to drive and use machines, contraindications, adverse drug reactions, drug-drug interactions, drug-food interaction, pregnancy considerations, lactation considerations, pediatric considerations, geriatric considerations, instructions to convert tablets or capsules into liquid forms, possibility of crushing and mixing with food or beverages, possibility of tablet splitting, pharmacokinetic information, shelf life, storage, name and address of manufacturers/ distributors, date of last revision, and references. DILs were evaluated and the scoring method in our study was modified according to the previous literature [3, 5, 16, 20, 29]. Each product’s information was scored either as 1 or as 0 (if present or absent respectively). The total of these scores was calculated for both local and imported products separately, and then compared. The second method was manual word-counting [5, 20, 29, 30]. Twenty-three of the 31 statements that were available in both local and imported DILs were also evaluated in terms of total word-counts: the median (interquartile range) word-count for each statement was determined separately for the two groups and compared. For the remaining 8 statements this was not performed, either because they were not present in any leaflet, or because counting the number of words would not be meaningful.

Two Pharm. Ds′ authors (Dina Arandy and Maysa Abu-Hashia) reviewed all the DILs independently for extracting data, and summarizing findings. Disagreements in the selection of criteria or substance were resolved by consensus-based discussion and then assessed by a third reviewer (Bahaa Al-hroub).

### Statistical analysis

The data were entered and analyzed by using Statistical Package for Social Sciences (SPSS) Version 16. Descriptive results were expressed as frequency and percentage, or/and median and interquartile range (IQR). Comparison of the word-counts between the local and imported drugs was by Mann-Whitney U-test. A *p* value of < 0.05 was considered significant.

## Results

Thirty-five NSAID package inserts that are available in the Palestinian marketplace were analyzed based on 31 criteria. As previously noted, 18 (51%) were local products and 17 (49%) were imported. The 18 locally-produced agents are manufactured by 4 Palestinian pharmaceutical companies specified as A (7 products), B (5 products), C (5 products) and D (one product). Upon analysis, and as Table 2 illustrated, we found that all DILs (100%) mentioned brand name, active ingredient, indications, direction for use, contraindications, adverse drug reactions, drug-drug interactions, name and address of manufacturer, pregnancy considerations and storage. Most DILs had information regarding therapeutic class (89%), overdose and management (97%), warnings and precautions (97%), lactation considerations (97%) and shelf life (97%). Information regarding missing dose, maximum dose, mechanism of action and effect on ability to drive and use machines presented in 96, 77, 17, and 71% of all DILs respectively. Information regarding pharmacokinetics, date of last revision, inactive ingredients and instructions to convert tablet or capsule into liquid forms were not mentioned in any local product, whereas the ‘references’ category was not used by any imported product. In general, the number of local leaflets that met the listed criteria was lower than the number of the imported ones.

**Table 2:** Scores of the thirty one statements written in the leaflets inserted in the local and imported NSAIDs

Local Companies								
No.	Criteria	A (*N* = 7)	B (*N* = 5)	C (*N* = 5)	D (*N* = 1)	Total scores of Local products (*N* = 18)	Total scores of Imported products (*N* = 17)	Total (*N* = 35)
1.	Brand name	7	5	5	1	18 (100)	17 (100)	35 (100)
2.	Active ingredient	7	5	5	1	18 (100)	17 (100)	35 (100)
3.	Inactive ingredients (excipients)	0	0	0	0	0 (0)	17 (100)	17 (49)
4.	Therapeutic class	4	4	5	1	14 (78)	17 (100)	31 (89)
5.	Mechanism of action	0	1	2	0	3 (17)	3 (18)	6 (17)
6.	Indications	7	5	5	1	18 (100)	17 (100)	35 (100)
7.	Drug dose	5	3	4	1	13 (72)	14 (82)	27 (77)
8.	Duration of using	2	0	2	1	5 (28)	12 (71)	17 (49)
9.	Missing dose	7	4	1	1	13 (72)	11 (65)	24 (69)
10.	Maximum dose	5	2	5	1	13 (72)	14 (82)	27 (77)
11.	Directions for use	7	5	5	1	18 (100)	17 (100)	35 (100)
12.	Overdose and management	7	4	5	1	17 (94)	17 (100)	34 (97)
13.	Warning and precautions	7	4	5	1	17 (94)	17 (100)	34 (97)
14.	Effect on ability to drive and use machines	4	3	2	0	9 (50)	16 (94)	25 (71)
15.	Contraindications	7	5	5	1	18 (100)	17 (100)	35 (100)
16.	Adverse drug reactions	7	5	5	1	18 (100)	17 (100)	35 (100)
17.	Drug-drug interactions	7	5	5	1	18 (100)	17 (100)	35 (100)
18.	Drug-food interactions	2	3	0	0	5 (28)	13 (76)	18 (51)
19.	Pregnancy considerations	7	5	5	1	18 (100)	17 (100)	35 (100)
20.	Lactation considerations	7	4	5	1	17 (94)	17 (100)	34 (97)
21.	Pediatric considerations	7	5	5	1	18 (100)	17 (100)	35 (100)
22.	Geriatric considerations	2	0	1	0	3 (17)	15 (88)	18 (51)
23.	Possibility of tablet splitting	0	1	0	0	1 (6)	11 (65)	12 (34)
24.	Possibility of crushing and mixing with food or beverages	7	2	1	1	11 (61)	15 (88)	26 (74)
25.	Instructions to convert tablets or capsules into liquid forms	0	0	0	0	0 (0)	1 (6)	1 (3)
26.	Pharmacokinetic information	0	0	0	0	0 (0)	2 (12)	2 (6)
27.	Shelf life	7	5	5	1	18 (100)	16 (94)	34 (97)
28.	Storage	7	5	5	1	18 (100)	17 (100)	35 (100)
29.	Name and address of manufacturers/distributors	7	5	5	1	18 (100)	17 (100)	35 (100)
30.	Date of last revision	0	0	0	0	0 (0)	13 (76)	13 (37)
31.	References	7	2	0	0	9 (50)	0 (0)	9 (26)

As for word-counting, 12 categories out of the 23 selected criteria were significantly different between the two studied agents. As Table 3 shows, significant differences were found in the following criteria and were more likely to be higher in imported agents: “therapeutic class” (*p*-value = 0.026), “warnings and precautions” (*p*-value = 0.002), “effects on ability to drive and use machines” (*p*-value = 0.002), “contraindications” (*p*-value = 0.005), “adverse drug reactions” (*p*-value = 0.001), and “drug-drug interactions” (*p*-value = 0.001).

**Table 3:** Statistical difference between word-counts for local and imported NSAID for all products combined

Variable	local Median [Q1-Q3]	Imported Median [Q1-Q3]	*P* value	Fold difference in medians
Therapeutic class	2.60 [2.00–4.50]	4.00 [4.00–8.50]	0.026	1.53
Mechanism of action	0.00 [0.00–4.25]	0.00 [0.00–17.00]	0.713	1
Indications	30.60 [19.50–42.75]	39.80 [18.50–74.50]	0.566	1.30
Drug dose	21.50 [12.50–51.25]	44.30 [28.30–65.25]	0.233	2.06
Duration of using	1.60 [0.00–5.25]	20.00 [2.35–27.60]	0.059	12.5
Missing dose	21.00 [16.65–32.75]	22.50 [0.00–28.40]	0.507	1.07
Maximum dose	6.00 [1.00–9.25]	7.00 [6.00–28.80]	0.169	1.16
Directions for use	33.50 [31.00–52.00]	30.00 [22.75–45.85]	0.200	1.11
Overdose and management	36.00 [35.80–41.80]	55.00 [36.80–93.75]	0.144	1.52
Warning and precautions	66.00 [31.00–92.30]	261.00 [183.00–425.50]	0.002	3.95
Effect on ability to drive and use machines	11.00 [0.00–14.80]	33.00 [20.00–61.50]	0.002	3.00
Contraindications	29.00 [19.00–84.00]	132.00 [100.00–152.75]	0.005	4.55
Adverse drug reactions	76.50 [60.25–104.50]	327.00 [213.20–503.75]	0.001	4.27
Drug-drug interactions	53.00 [49.00–67.05]	180.00 [118.25–257.85]	0.001	3.39
Drug-food interactions	4.30 [0.00–7.50]	18.50 [4.35–30.50]	0.053	4.30
Pregnancy considerations	19.00 [13.75–27.25]	45.00 [11.25–88.55]	0.170	2.36
Lactation considerations	13.00 [11.65–24.25]	9.00 [8.75–28.15]	0.595	1.44
Pediatric considerations	48.00 [35.65–64.75]	87.00 [56.25–120.95]	0.024	1.81
Geriatric considerations	0.00 [0.00–4.15]	42.40 [21.50–71.85]	0.002	> 42.40
Possibility of tablet splitting	0.00 [0.00–0.00]	5.00 [0.00–13.00]	0.010	> 5.00
Possibility of crushing and mixing with food or beverages	2.60 [1.50–3.15]	4.40 [3.00–6.10]	0.030	1.69
Shelf life	15.00 [10.60–16.00]	21.00 [16.35–27.00]	0.012	1.40
Storage	18.00 [14.50–32.00]	58.00 [43.85–78.05]	0.005	3.22

In addition, the difference between the two studied categories was significant in word-count of the categories “pediatric considerations” (*p*-value = 0.024), “geriatric considerations” (*p*-value = 0.002), “possibility of tablet splitting” (*p*-value = 0.010), “possibility of crushing and mixing with food and beverages” (*p*-value = 0.030), “shelf life” (*p*-value = 0.012) and “storage” (*p*-value = 0.005); (Table 3).

As for the “geriatric considerations” category, the median word-count of local DILs was 0.00 [0.00–4.15] words against 42.40 [21.50–71.85] words for the imported, which is a >  42.4-fold difference, whereas the median word-count of local DILs was 15.00 [10.60–16.00] words against 21.00 [16.35–27.00] words for the “shelf life” category, which is 1.4-fold difference. For other categories that have significant *P* values, 1.5–5-fold difference was found (Table 3).

## Discussion

In the current study, Palestinian DILs of NSAIDs were analyzed and evaluated to assess if they provide relevant information concerning safe and proper medication use. Both the quality and scope of DILs’ information are important for patients, health care professionals and others i.e.: medical students.

The results of our study indicate that there are many information deficiencies in DILs and these should be addressed. In our study, “duration of using” was mentioned in about half of the DILs and these results seem to be consistent with other research conducted in Saudi Arabia, which found that about 50% of DILs mentioned this [31]. Inclusion of a “date of last revision” is desirable as this will increase both the physician and patient’s trust toward the prescribed drug. It is interesting to note that most DILs in our study mentioned adverse drug reactions, warning and precautions, and this encourages the rational use of drugs. The “shelf life” of the drug is of obvious importance and is mentioned in the majority of DILs in our study. While observing the shelf life will not necessarily affect the safety of the drug, it can possibly influence the efficacy and quality of the drug, resulting in a poor control of the disease. What is surprising is that “inactive ingredients” are not mentioned in any DILs of Palestinian-produced products, but are noted in all imported products. These results match those observed in another study performed in our country [5]. Inactive ingredients such as artificial sweeteners, sodium salts and others should be listed in DILs. These ingredients may affect certain patient conditions or diseases [32, 33], i.e. sodium salts can affect blood pressure in patients with hypertension, and some patients may in fact be allergic to those ingredients. Thus, if inactive ingredients go unmentioned, it could expose a patient to a dangerous event that leads to hospitalization or even death.

The most controversial finding to emerge from the analysis of word-count is that the related sections of the most criteria were more fully expressed by imported leaflets, compared with the local ones. Of course, increased text volume can alter leaflet design in terms of font size and other characteristics, and this may affect both the readability and the patient’s motivation to read medication leaflets. Fuchs and his colleagues showed that small font sizes and the paper size itself is more likely with increased text volume, and this makes it harder for patients to locate product medical information, affecting their confidence to take the required medicines [34]. A high information volume in leaflets can also affect patients’ comprehensibility and perceptions of correct medication instructions. Two studies revealed that the increase in DIL length guaranteed a higher incidence of long sentences, non-quantifiable phrases, or even difficult words; this hindered the desire of concise and essential information for both patients and healthcare providers [35, 36]. Also, according to many guidelines, information about overdose should be clear and concise, to avoid confusing patients and making them concerned about their ability to follow instructions [37–39].

DILs with high information volume meet the criteria of ample content and can protect the drug’s manufacturer legally, but this can be distracting to many patients at the same time, affecting the attainability of adequate information to achieve the desired therapeutic outcomes [2]. In an American survey, prescriptions for Metformin and Lisinopril were filled by professional shoppers in a sample of 365 nationwide pharmacies, with consumer medication information leaflets that differed considerably in word-count. The range was 33-to-2482 words and this study found that leaflets with higher information volume, that got better scores of content quality, but failed to achieve the intended purpose of how patients can get optimal benefits from therapy [40].

As for the “side effects” section, complex and long lists of potential adverse reactions are considered to be “over warning” and result in overloaded information, diminishing labeling effectiveness. This issue was highlighted by the US Food and Drug Administration (FDA) as a new labeling guideline in 2006, that discourages the inclusion of adverse events lists which are exhausted in spite of frequency and minority [41]. In 2015, the FDA stated that adverse reactions which are considered as “less common” should be present in medication leaflets only if they are linked to medications by a causal relationship [42]. Herber and his coworkers stated that the high amount of mentioned drug interactions and side effects in package inserts provokes different emotional reactions and leads to different patient behaviors toward taking medications [43].

This is the first comparative study that evaluates DILs of both local and imported NSAIDs in the Palestinian West Bank market; one of its expected results is to stimulate more research into the evaluation of locally-produced DILs. The present study had the following limitations: First, the number of package inserts included in the study was small; for some active ingredients, only one local product and one imported product were included. Second, the study covered only one group of medication (NSAIDs) and included only such drugs as were manufactured by both local and imported companies, and only the oral solid dosage forms were included. Finally, this study has been focused on the bulk and availability of key information, and did not evaluate the readability or the comprehensibility of DILs. Therefore, studies that focus on these issues are required.

## Conclusions

The present study showed that both the quality and the quantity of DILs provided information of Palestine-produced NSAIDs is significantly different compared with its imported equivalents to be found in the Palestinian market. Incomplete information about contraindications, interactions, warnings and precautions affect the safety and efficacy of medication use, so to avoid any medication errors that may be a result from information deficits in local DILs, we recommend that appropriate measures should indeed be implemented by the Palestinian Ministry of Health and local manufacturers, to determine the key information, and to make an improvement in the existing DILs, based on best practice. This will help improve the quality and fulsomeness in the DILs of local NSAIDs and make them complete, up to date, and meet the needs of the Palestinian patients.

## Data Availability

The raw data supporting the findings presented in this study will be available from the corresponding author upon request.

## Abbreviations

DILs: Drug information leaflets
FDA: Food and Drug Administration
IQR: Interquartile range
IRB: Instutional Review Board
MOH: Ministry of Health
NSAIDs: Non-steroidal anti-inflammatory drugs
OTC: Over-the-counter
SD: Standard deviation
SPSS: Statistical package for social sciences
